# The Emergence of HIV Transmitted Resistance in Botswana: “When Will the WHO Detection Threshold Be Exceeded?”

**DOI:** 10.1371/journal.pone.0000152

**Published:** 2007-01-17

**Authors:** Raffaele Vardavas, Sally Blower

**Affiliations:** Semel Institute of Neuroscience and Human Behavior, David Geffen School of Medicine at University of California Los Angeles, Los Angeles, California, United States of America; London School of Hygiene and Tropical Medicine, Peru

## Abstract

**Background:**

The Botswana antiretroviral program began in 2002 and currently treats 42,000 patients, with a goal of treating 85,000 by 2009. The World Health Organization (WHO) has begun to implement a surveillance system for detecting transmitted resistance that exceeds a threshold of 5%. However, the WHO has not determined when this threshold will be reached. Here we model the Botswana government's treatment plan and predict, to 2009, the likely stochastic evolution of transmitted resistance.

**Methods:**

We developed a model of the stochastic evolution of drug-resistant strains and formulated a birth-death Master equation. We analyzed this equation to obtain an analytical solution of the probabilistic evolutionary trajectory for transmitted resistance, and used treatment and demographic data from Botswana. We determined the temporal dynamics of transmitted resistance as a function of: (i) the transmissibility (i.e., fitness) of the drug-resistant strains that may evolve and (ii) the rate of acquired resistance.

**Results:**

Transmitted resistance in Botswana will be unlikely to exceed the WHO's threshold by 2009 even if the rate of acquired resistance is high and the strains that evolve are half as fit as the wild-type strains. However, we also found that transmission of drug-resistant strains in Botswana could increase to ∼15% by 2009 if the drug-resistant strains that evolve are as fit as the wild-type strains.

**Conclusions:**

Transmitted resistance will only be detected by the WHO (by 2009) if the strains that evolve are extremely fit and acquired resistance is high. Initially after a treatment program is begun a threshold lower than 5% should be used; and we advise that predictions should be made before setting a threshold. Our results indicate that it may be several years before the WHO's surveillance system is likely to detect transmitted resistance in other resource-poor countries that have significantly less ambitious treatment programs than Botswana.

## Introduction

Antiretroviral therapy (ART) has drastically reduced mortality and morbidity of HIV/AIDS in resource-rich countries; however there are now fairly high levels of drug resistance in many of these countries [1]. Therefore there is concern that the rollout of ART in Africa could quickly lead to high levels of transmitted resistance arising which would reduce the effectiveness of epidemic control efforts. The World Health Organization (WHO) has developed a surveillance system for monitoring the emergence of transmitted resistance in resource-poor countries [2], [3]. The WHO detection methodology is based upon a binomial sequential lot quality assurance (BSLQA) sampling method with an arbitrary detection threshold of 5% [4], [5]. This threshold enables determination of whether the incidence of transmitted resistance lies in one of three categories: less than 5%, 5% to 15% or over 15%. The range of the minimal number of newly infected treatment-naïve adults that need to be sampled to detect a 5% threshold ranges from 50 to 70 newly infected treatment-naïve adults [2], [3]. Hence the BSLQA methodology is cost-effective and easy to implement, but will not be able to detect transmitted resistance until it has exceeded 5% of the incidence rate. It is unknown when the 5% threshold will be reached in any country, because the WHO has not made any country-specific predictions to determine how quickly transmitted resistance is likely to evolve. Here we develop a mathematical model that can be used to make short-term predictions for the emergence and stochastic evolution of transmitted resistance in a resource-poor country. We use our model to determine when the WHO's 5% threshold is likely to be exceeded in Botswana.

Botswana is one of the world's worst hit countries by the AIDS pandemic. An estimated 39% of Botswana's 730,000 adults between the ages of 15 and 49 are HIV-infected [6]. In 2002 HIV/AIDS was declared the most serious threat to the country, and their ART program was initiated. Currently the program treats 42,000 patients [7]. The goal is to treat 85,000 patients (or equivalently 30% of their HIV-infected individuals) by 2009 [8]. Botswana has by far the highest treatment rate in Africa, and has already slightly exceeded their scheduled annual treatment targets. Botswana is now considered a test nation by foreign investors for ART programs in sub-Saharan Africa. Recent substantial financial investments have enabled the opening of 31 treatment centers, ensuring that Botswana is quite likely to reach the government treatment goal by 2009. We use our stochastic model to predict the expected magnitude, and the temporal dynamics, of transmitted resistance in Botswana by modeling their government treatment plan. Specifically, we predict the likely evolutionary trajectory of transmitted resistance (due to the Botswana government's treatment plan) by varying: (i) the rate of acquired resistance, and (ii) the relative transmissibility (i.e., fitness) of resistant strains. We conclude by discussing the implications of our results in the context of the WHO's surveillance system for transmitted resistance.

The first-line drug regimen used in Botswana is a fixed combination of Zidovudine, Lamivudine, and Nevirapine or Efavirenz [9]. The evolution of transmitted resistance is driven by three key parameters: the treatment rate, the rate of development of acquired resistance and the fitness of the drug-resistant strains that evolve [1], [10]. We modeled the specific treatment rate for Botswana (see **Materials & Methods and Mathematical Details S1 section 1**). Thus, we investigated the effect of the other two key parameters on the evolution of transmitted resistance in Botswana. The rate of acquired resistance depends upon adherence, viral replication rate, and the specific mutations that arise [1]. Acquisition of one mutation will not necessarily reduce the clinical efficacy of a regimen; thus we did not model the accumulation of mutations, we modeled the virological failure rate due to acquired resistance. Under perfect adherence, only 5–10% of patients are likely to develop drug resistance in the first year [11]. However, if adherence is less than 100%, a greater proportion of patients will develop drug resistance within a year [12]. Recent studies on adherence in sub-Saharan Africa indicate that very favorable adherence rates can be reached and that these adherence rates can often be higher than those reported from North America: 77% adherence rates in Africa compared to 55% adherence rates in North America [13]. However patients in Botswana may suffer from poor treatment accessibility and interruption of drug supply which could compromise their adherence to treatment [14]; hence it is possible that rates of acquired resistance in Botswana could be high. In order to make worst case predictions, and to maximize the speed at which transmitted resistance could arise, we modeled high rates of acquired resistance. To make our short-term predictions we assumed that (on average) either 20% or 33% of treated patients could develop acquired resistance per year [15]–[19]. By considering the worst case scenarios for the rates of acquiring resistance we were able to determine the earliest times that transmitted resistance could reach the 5% detection threshold.

Patients who acquire resistance have the potential to transmit resistant strains via sexual transmission. Relatively little is known about the fitness of resistant strains of HIV *in vivo*. However, *in vitro* experiments have shown that the replication rates of resistant strains are generally less than that of wild-type strains [20]–[22]. Thus, it has often been assumed that resistant strains will always be less transmissible than wild-type strains. However, certain mutations may substantially reduce fitness whereas other mutations may have little effect. Hence it is possible that some drug-resistant strains could be almost as transmissible as wild-type strains. To consider all possibilities we examined the potential impact on transmitted resistance of a wide variety of mutations by varying the fitness of drug-resistant strains relative to wild-type strains from 0% to 75%. Specifically, we modeled the potential impact on transmitted resistance of drug-resistant strains with a relative fitness of: (i) 100%, (ii) 50%, or (iii) 25% [23]. The parameter values of the transmissibility/fitness of the wild-type strains and of the drug-resistant strains were calculated based upon the viral load of the treatment regimen (i.e., Zidovudine, Lamivudine, and Nevirapine or Efavirenz [9]) that is used in Botswana (see **Mathematical Details S1 section 1.2** for further discussion of calculation of these parameters). It should be noted that resistant strains that have low fitness may acquire compensatory mutations and hence increase their fitness over the long-term. Since our predictions are only short-term (i.e., only to 2009) we ignored the evolution of fitness.

There are relatively few mathematical models of ART and drug resistance; notably the first was in 2000 by Blower *et al.* [24]. Since then other studies have presented similar models [25], [26]. All three of these models have been based on deterministic formulations of the dynamics of HIV resistance. However, the early stages of the dynamics of an ART program are subject to large variability and therefore necessitate a stochastic treatment. In the analysis presented in this manuscript we are the first to develop a stochastic model of the evolution of drug resistance. We made projections for transmitted resistance in Botswana by first formulating a continuous-time Markov chain model; see **Materials & Methods** for mathematical details. To construct this model we developed a combination of deterministic and stochastic equations (see **Mathematical Details S1 sections 1 & 2** for detailed description and discussion). Deterministic equations were used to predict the dynamics of the number of adults in Botswana who are: (i) susceptible or (ii) infected with wild-type strains of HIV and treatment-naïve or (iii) infected with wild-type strains of HIV and on treatment. Their values are relatively large and currently are approximately 460,000 and 280,000 and 34,000 respectively. Thus, relative stochastic fluctuations in these three groups could be neglected and the mean-field dynamics from the deterministic version of our stochastic model used. However ART has only been available in Botswana since 2002 therefore the number of adults infected with drug-resistant strains is relatively small and quantifying the stochastic fluctuations in transmitted incidence is important. Essentially we are predicting the initial stage of an epidemic; specifically, the early stage of a drug-resistant HIV epidemic. At this early stage the population dynamics of transmitted resistance will be subject to a high degree of random (i.e., stochastic) fluctuations^1^. Capturing the effect of these stochastic fluctuations is an important part of any predictions made of the early stages of an epidemic. Therefore, in addition to mean-field predictions of transmitted resistance we also predicted the dynamics of the variance and skewness of these stochastic fluctuations by formulating and using a stochastic dynamic version of the deterministic model (see **Mathematical Details S1 section 2.1**). We derived a birth-death-immigration Master equation from our stochastic dynamic model, solved the Master equation analytically and used this analytical solution to determine explicit expressions for the mean, variance and skewness of the evolutionary dynamics [27]–[29] (see **Mathematical Details S1 section 2.3**). We then used these explicit expressions to predict the stochastic evolutionary trajectory, to 2009, of transmitted drug-resistant strains in Botswana. This approach is valid over the short-term dynamics. We chose not to simulate the process numerically via Monte Carlo simulations as our aim was to obtain expressions from which one can directly obtain the magnitude of the fluctuation [30].

## Results

We constructed the stochastic model by first formulating a deterministic version of the model and determining the mean-field dynamics. We then developed a stochastic version of the deterministic model and obtained the Master equation of the processes (see equation 1):
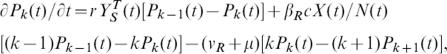
where *P_k_(t)* represents the probability that at time *t* the prevalence of drug-resistant HIV is *k*. The Master equation consists of three terms: the first term represents the process by which individuals acquire resistance; the second term represents the process whereby individuals transmit drug-resistant strains and the last term represents the process whereby individuals develop AIDS or die in the process of developing AIDS. This Master equation was reformulated in terms of a cumulant generating function. By solving the latter equation we obtained a solution that provided the time dynamics of the central moments of the probability distribution *P_k_(t)*. We retained the first three central moments, and therefore obtained the dynamics of the mean, variance and skewness of the prevalence of transmitted resistance. Finally, we used this solution to obtain the generating function of the incidence and therefore also obtained the dynamics of the mean, variance and skewness of the incidence of transmitted resistance (see **Mathematical Details S1 section 2.4**). We used these solutions to generate our projections of the likely evolutionary trajectory of transmitted resistance that is to be expected as a result of the Botswana governments' treatment plan.

In order to solve the Master Equation we had to make the approximation that the proportion of the adults that were uninfected remained constant throughout the time frame of our predictions. To test this approximation we compared the mean-field predictions obtained by solving the Master equation to those obtained by the numerical integration of the deterministic version of the model for the duration of interest (i.e., up to 2009). We found that the percentage error made by the mean-field solution of the Master equation was only 6% in absolute values by 2009; this 6% error translates into an overestimation (during the last quarter of 2008) of the cumulative incidence of transmitted resistance by ∼74 individuals out of a total number of 1230^2^.

**Figure 1:**
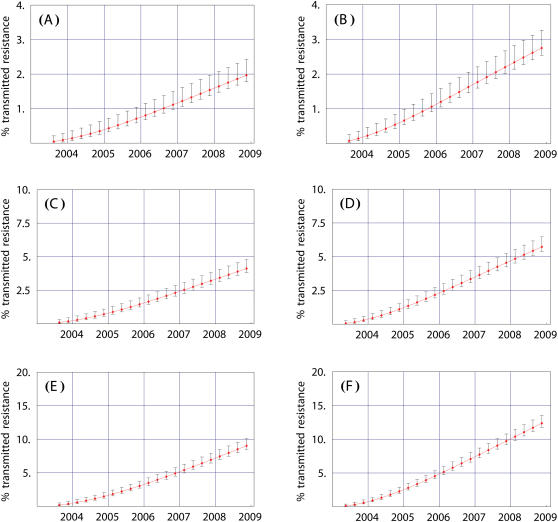
Predictions using the cumulant generating function showing quarter yearly expected percentage values of transmitted resistance due to the Botswana's government treatment plan. The vertical bars represent the possible range of values due to the stochastic fluctuation contained in one standard deviation over the expected percentage. Here, we have assumed that the average progression time to AIDS for HIV infected patients that are treatment-naïve, treated drug-sensitive and drug-resistant are 10, 18 and 12 years respectively [23], [33]; however since our predictions are only for three years our results are robust with respect to this assumption. The untreated and treated drug-sensitive transmissibility coefficient per partnership were calculated, on the basis of the Botswana treatment regimen of a fixed combination of Zidovudine, Lamivudine, and Nevirapine or Efavirenz, to be 0.12 and 0.04 respectively [31], [34], [35] (see **Mathematical Details S1 section 1.2** to see how the values of these parameter estimates were derived). The patient population has an average annual probability of developing drug resistance specified by the parameter *r*, and the transmissibility/fitness of drug-resistant strains is specified by the parameter β*_R_* (see **Mathematical Details S1 section 1.2** to see how the values of these parameter estimates were derived). The six panels show predictions using the following parameter sets: (A) *r^−1^* = 5 years, β*_R_* = 0.03 (B) *r^−1^* = 3 years, β*_R_* = 0.03 (C) *r^−1^* = 5 years, β*_R_* = 0.06 (D) *r^−1^* = 3 year, β*_R_* = 0.06 (E) *r^−1^* = 5 years, β*_R_* = 0.12 (F) *r^−1^* = 3 years, β*_R_* = 0.12.

By using the generating function for cumulative incidence over periods of 3 months we determined that whether the WHO surveillance threshold of 5% is likely to be exceeded by 2009 depends critically upon both the fitness of the drug-resistant strains and the rate of development of acquired resistance. Data shown in Fig. 1 are predictions obtained by using the analytical expression for the mean transmitted incidence of the drug-resistant strains and are expressed as a fraction of the total incidence for both wild-type and drug-resistant strains (see **Mathematical Details S1 section 2.3 & 2.4**). The vertical bars show the corresponding expected probabilistic fluctuation ranges, within one standard deviation, due to the stochasticity in the dynamics. These probabilistic ranges were obtained using both the expressions for the variance and skewness in the cumulative incidence over 3 month periods (see **Mathematical Details S1 section 2.4**). Panels (1A), (1C) and (1E) show probabilistic predictions when we assumed that (on average) 20% of the patients would acquire drug resistance per year (i.e., that on average all of the patients would develop resistance in 5 years). Panels (1B), (1D) and (1F) show probabilistic predictions when we assumed that 33% of the patients would acquire drug resistance per year (i.e., that, on average, all of the patients would develop resistance in 3 years). The relative fitness of the drug-resistant strains with respect to the wild-type strains that are used in the calculations are: (i) 25% (Fig. 1A) and (Fig. 1B), (ii) 50% (Fig. 1C) and (Fig. 1D), and (iii) 100% (Fig. 1E) and (Fig. 1F).

Our results show that if the drug-resistant strains that evolve are only 25% as fit as the wild-type strains then transmitted resistance will reach only ∼3% in the next three years (Fig. 1A). Thus the WHO surveillance threshold is not likely to be exceeded by 2009, even if the rate of acquired resistance is very high (Fig. 1B). Transmitted resistance will reach 6% by 2009 if the drug-resistant strains that evolve are half as fit as the wild-type strains and the rate of acquired resistance is very high (Fig. 1D). Thus, under these conditions, the WHO surveillance threshold is likely to be exceeded. If the drug-resistant strains that evolve are half as fit as the wild-type strains, but the rate of development of acquired resistance is only 20% per year, transmitted resistance will only rise to just below 5% by 2009 (Fig. 1C). Hence, under these conditions, the WHO surveillance threshold may not be exceeded. The expected probabilistic fluctuations are large enough that there is a fair probability that transmitted drug resistance could rise to just above 5% by 2009, and thus exceed the threshold. Finally, if the drug-resistant strains that emerge are as fit as wild-type strains then the threshold will be reached in 2006 (Fig. 1F) or 2007 (Fig. 1E), and levels of transmitted resistance could be as high as 13% by 2009 (Fig. 1F). Obviously, if drug-resistant strains evolve that are more fit than wild-type strains the levels of transmitted resistance will be even higher by 2009 (results not shown). It is also possible that transmitted resistance may not be observed at all if a more suppressive treatment regimen is used than is currently planned.

## Discussion

We have used a fairly simple stochastic model to predict, to 2009, the evolutionary trajectory for the incidence of transmitted resistance that is to be expected to occur in Botswana as a result of the government's treatment plan. Our model includes the principle processes that are involved in the short-term dynamics of the emergence of drug resistance in a sub-Saharan African country once a treatment plan has been initiated. It has sometimes been argued, on the basis of a high viral load, that primary infection maybe responsible for a large percentage of infections [31]. However, the length of time spent in the primary infection stage is substantially shorter (i.e., only a couple of months) than the length of time that is spent in the later stages of HIV infection which is generally many years before developing AIDS. Consequently, due to the differential length of time in these stages, the number of individuals that are in the primary infection stage at any one time are substantially fewer than the number of individuals that are in the later (but still untreated) stages of HIV infection. Consequently we did not deem it necessary to model explicitly the effect of primary infection as we were making only short-term predictions. A more detailed model that includes additional processes such as reversion of drug-resistant strains, the effects of super-infection and primary infection should be developed if longer-term predictions are to be made. However, it should be noted that for short-term predictions the contribution of such processes has fairly minimal effects. Generally, all processes that have time scales much larger than the time frame chosen for making short-term predictions can be safely ignored. Nor, did we include the process of vertical transmission of drug resistance. Our model captures only the dynamics of transmitted resistance that have occurred due to sexual transmission, because this is the type of transmitted resistance that will be monitored in Botswana by the WHO's surveillance system.

It is possible that HIV infected individuals will obtain ART outside the official treatment program. This could potentially increase levels of transmitted resistance. Since Botswana plans to treat, by 2009, a very high percentage (∼30%) of HIV infected individuals the number of HIV-infected individuals who will need to obtain drugs outside the program is likely to be fairly small. Thus, any additional drug resistance generated by these individuals is likely to make only a small contribution to the incidence rate. We stress that the analytical expressions that we have formulated are only valid for short-term dynamics (i.e., a few years) when the proportion of the population that is uninfected and the proportion that is infected with wild-type and treatment-naïve stay approximately at their equilibrium values. The approximation of equilibrium is appropriate for Botswana over the next few years because: (i) the population infected with wild-type HIV and treatment-naïve is very large, currently approximately 280,000 adults, and (ii) and prevalence is approximately stable at 40% (the prevalence is stable as the death rate plus the rate of adults that develop AIDS for in Botswana has reached a high level and approximately balances the incidence rate). Use of our analytical expressions beyond short-term dynamics would be inappropriate because of the assumptions that have been made. Furthermore, in the regime beyond short-term dynamics, stochastic effects are increasingly less important and therefore often stochastic models are unnecessary to predict long-term dynamics.

Botswana will have achieved high treatment rates by 2009. Our results imply that, unless the drug-resistant strains that evolve in Botswana are extremely transmissible, the WHO threshold for detection of transmitted resistance is unlikely to be exceeded by 2009. Although the BSLQA method using a threshold value of 5% requires a small sample size and is relatively inexpensive it may actually not be cost-effective at the early stages of the treatment program. Therefore, it may be appropriate to consider using a lower threshold value depending on mathematical predictions of transmitted resistance dynamics. Our predictions show that checking for transmitted resistance in Botswana in early 2007 using a lower threshold value of ∼3% would provide a more accurate representation of the present situation. If transmitted resistance is found to be at or above 3% then repeating the BSLQA test in the next scheduled occasion using a 5% threshold value would provide more information as to how quickly transmitted resistance is increasing in Botswana. Although this would be more expensive, it would probably be more cost-effective than the current strategy.

We stress that other models should be constructed and their results compared with our predictions. We propose that the WHO's surveillance system should be designed on the basis of quantitative predictions and should not be based upon specification of arbitrary thresholds. We advise that, at the beginning of treatment programs in resource-poor countries, that the detection threshold should be lower than 5% in order to detect transmitted resistance. A lower threshold would require a larger sample size and therefore would be more expensive, although potentially more effective. We recommend that WHO surveillance should be initiated in Botswana, and in resource-poor countries, only when transmitted resistance is expected to exceed the threshold. However, sentinel sites for surveillance should be used throughout the rollout [1]. We also suggest that quantitative predictions for transmitted resistance should be made for other countries where the rollout of ART is just beginning. We conclude that it is likely that, if the 5% threshold is used, it could be many years before the WHO will detect transmitted resistance in other resource-poor countries that have less ambitious treatment programs than Botswana.

## Materials and Methods

To predict the stochastic evolution of transmitted resistance we developed a continuous time Markov chain model; see **Mathematical Details S1**, a flow diagram and the model equations for both the deterministic (**section 1.1**) and stochastic version of the model (**section 2**). The **Mathematical Details S1** also includes parameter definitions and estimations (**section 1.2**), initial conditions (**section 1.3**) and a comparison between the deterministic and the stochastic model (**section 2.5**).

**Figure 2:**
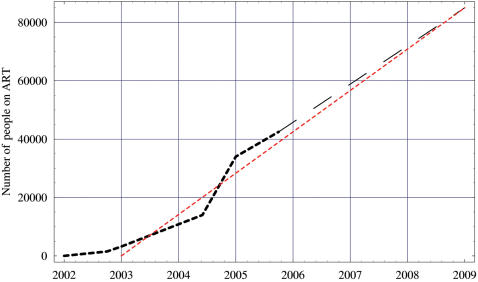
Empirical data from the Botswana's government treatment program are shown by the filled boxes. The aim of the Botswana ART program is to reach 85,000 patients by 2009 (black dashed line) [8]. The treatment used by our model uses the linear fit shown by the dashed gray line, this gives a constant per capita treatment rate of 0.050 per year.

We modeled the stochastic dynamics of the emergence (by acquired resistance) and the evolutionary trajectory of transmitted resistance after the introduction of ART at the beginning of the Botswana treatment program to 2009. Our stochastic Markov chain model captures the essential processes of HIV transmission dynamics (see **Mathematical Details S1** for details of mathematical structure). Each year a proportion of the drug-naïve HIV-infected group initiates treatment, a proportion of the treated group discontinues treatment, and a proportion of the treated group develops acquired resistance [32]. We calculated the treatment rate from the projected number of patients that the Botswana ART program plans to treat over the next three years. The program plans to treat 85,000 patients by 2009 [8] (Figure 2). The treatment rate was found by assuming that it remains constant such that by the year 2009 Botswana will have 85,000 patients that have received ART; this is the planned treatment goal of the Botswana government. Our modeled treatment rate (dashed gray line) and the actual data from 2002 to 2005 (dashed red line) are extremely close and are shown in Figure 2. Treatment benefits were modeled, in treated patients in comparison with untreated HIV-infected individuals, by increasing life expectancy (1/*v_S_^T^>1/v_S_^U^*) [12] (see **Mathematical Details S1 section 1.2** for parameter definitions) and by reducing viral load (and hence transmissibility/fitness: β*_S_^U^*>β*_S_^T^*). The patient group has an average annual probability of developing drug resistance specified by the parameter *r*. Treated individuals that develop drug resistance increase their viral load and progress to AIDS faster than treated individuals infected with wild-type strains (1/*v_S_^T^>1/v_R_*); however it should be noted that since our predictions are only for three years our results are robust with respect to this assumption. Individuals infected with drug-resistant strains can transmit these strains; the transmissibility/fitness of drug-resistant strains is specified by β*_R_* (see **Mathematical Details S1 section 1.2** for how this parameter was calculated).

## Supporting Information

Mathematical Details S1 Here we present technical details that describe mathematical methodologies and analytical derivations used for our results presented in the main text and parameter estimates. In section 1 we present our mathematical model, which describes the dynamics of the emergence of acquired and transmitted drug-resistant HIV in a population. This is followed by a discussion of the parameters used in the model. We formulate the ordinary differential equations (ODEs) describing the mean-field population dynamics and describe the initial conditions for the model. In section 2 we formulate the dynamics of the drug-resistant population using a Markov chain equation and we discuss the approximations required for this formulation to produce acceptably accurate dynamics. We then show how this can be used to obtain expressions for the evolution of the mean, variance and skewness of the cumulative transmitted resistance.
(0.17 MB PDF)

## Footnotes

1 It is the relative fluctuations that are large during the early stages of the epidemic. Indeed, the cumulative random fluctuations increase with time. However, these become progressively less important as a fraction of the prevalence. Therefore, as a fraction of the prevalence, random fluctuations are mostly important during the early stages of an epidemic.

2 This number was obtained by considering the worst case scenario using the highest rate of both acquired resistance and transmitted resistance that we have considered.
