# Supplementary material for: The Emergence of HIV Transmitted Resistance in Botswana: “When Will the WHO Detection Threshold Be Exceeded?”
Source: PLoS One. 2007 Jan 17;2(1):e152. doi: 10.1371/journal.pone.0000152 (PMC1764713; doi:10.1371/journal.pone.0000152)
Supplement: Mathematical Details S1 — Here we present technical details that describe mathematical methodologies and analytical derivations used for our results presented in the main text and parameter estimates. In section 1 we present our mathematical model, which describes the dynamics of the emergence of acquired and transmitted drug-resistant HIV in a population. This is followed by a discussion of the parameters used in the model. We formulate the ordinary differential equations (ODEs) describing the mean-field population dynamics and describe the initial conditions for the model. In section 2 we formulate the dynamics of the drug-resistant population using a Markov chain equation and we discuss the approximations required for this formulation to produce acceptably accurate dynamics. We then show how this can be used to obtain expressions for the evolution of the mean, variance and skewness of the cumulative transmitted resistance. (0.17 MB PDF) [file pone.0000152.s001.pdf]

# Mathematical Details S1:

## The emergence of HIV transmitted resistance in Botswana:

## "When will the WHO detection threshold be exceeded?"

Raffaele Vardavas and Sally Blower

Here we present technical details that describes mathematical methodologies and analytical derivations used for our results presented in the main text and parameter estimates. In section 1 we present our mathematical model which describes the dynamics of the emergence of acquired and transmitted drug-resistant HIV in a population. This is followed by a discussion of the parameters used in the model. We formulate the ordinary differential equations (ODEs) describing the mean-field population dynamics and describe the initial conditions for the model. In section 2 we formulate the dynamics of the drug-resistant population using a Markov chain equation and we discuss the approximations required for this formulation to produce acceptably accurate dynamics. We then show how this can be used to obtain expressions for the evolution of the mean, variance and skewness of the cumulative transmitted resistance.

## Contents

**1 The Deterministic model** **2**

1.1 The mean-field dynamics . . . . . . . . 2

1.2 The parameters: definition and estimation . . . . . . . . 3

1.3 The initial conditions . . . . . . . . 5

**2 Stochastic formulation for the drug-resistant population dynamics** **5**

2.1 Stochastic vs. Deterministic . . . . . . . . 5

2.2 The Master equation . . . . . . . . 6

2.3 Solution for the Mean, Variance and Skewness . . . . . . . . 7

2.4 Solution for the Cumulative Incidence . . . . . . . . 8

2.5 Solution of the Master Equation versus the numerical integration of the ODEs . . . . . . . . 8

# 1 The Deterministic model

## 1.1 The mean-field dynamics

In Fig.1 we show the flow diagram of the compartmental Markov chain model that we used to describe the dynamics of the emergence of acquired and transmitted drug-resistant HIV in a population. The model identifies four population groups: sexually-active susceptible adults $X$, treatment-naive adults infected with wild-type HIV strains $Y_S^U$, treated adults infected with wild-type HIV strains $Y_S^T$, and adults infected with drug-resistant HIV strains $Y_R$. The drug-resistant group could be subdivided into treated and treatment-naive classes. In the limit of large population numbers present in all four compartments, the dynamics

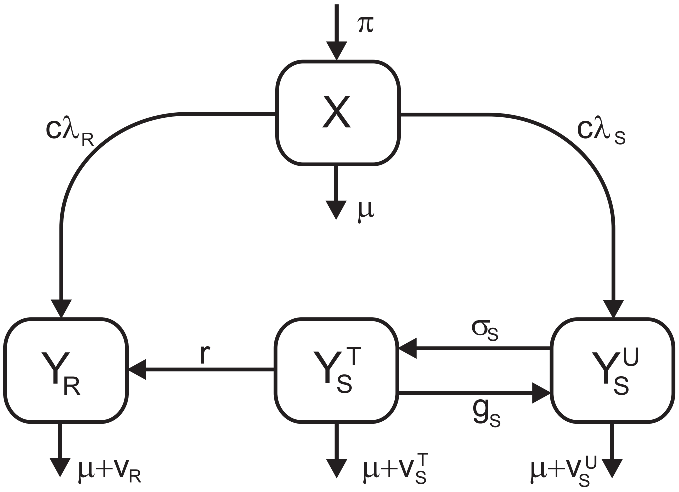


Figure 1: Flow diagram illustrating the transmission dynamics of an HIV epidemic in the presence of antiretroviral therapy (ART).

can be accurately described by mean-field equations. These equations neglect all stochastic aspects present in the Markov chain model and only focus on the evolution of mean population numbers. The mean-field population dynamics associated with the continuous time Markov chain compartmental model is described by the following ODEs:

$$\dot{X}(t) = \pi - \{c[\lambda_S(t) + \lambda_R(t)] + \mu\} X(t) , \tag{1.1}$$

$$\dot{Y}_S^U(t) = c\lambda_s(t)X(t) + g_s Y_S^T(t) - (\sigma_s + v_s^U + \mu)Y_S^U(t) , \tag{1.2}$$

$$\dot{Y}_S^T(t) = \sigma_s Y_S^U(t) - (g_s + r + v_s^T + \mu)Y_S^T(t) , \tag{1.3}$$

$$\dot{Y}_R(t) = rY_S^T(t) + c\lambda_R(t)X(t) - (v_R + \mu)Y_R(t) , \tag{1.4}$$

where

$$\lambda_S(t) = N^{-1}(t)[\beta_S^U Y_S^U(t) + \beta_S^T Y_S^T(t)]\,, \quad (1.5)$$

$$\lambda_R(t) = N^{-1}(t)\beta_R Y_R(t)\,, \quad (1.6)$$

$$N(t) = X(t) + Y_S^U(t) + Y_S^T(t) + Y_R(t)\,. \quad (1.7)$$

## 1.2 The parameters: definition and estimation

The parameters that appear in Eqs. (1.1)-(1.7) are defined as follows: $\pi$ is the inflow rate of newly sexually active adults; $\mu^{-1}$ is the average length of time an adult acquires new sex partners; $c$ is the average number of new sex partners a susceptible individual acquires per year; $\sigma_s$ is the per capita treatment rate per year; $g_s$ is the yearly proportion of patients suspending treatment and $r^{-1}$ is the average time required for a patient to develop drug resistance when on treatment. The disease progression rate to AIDS for the wild-type treatment-naive, wild-type treated and drug-resistant populations are respectively described by the parameters $v_S^U$, $v_S^T$ and $v_R$; the transmissibility (i.e., *fitness*) coefficients for the wild-type treatment-naive, wild-type treated and drug-resistant populations are respectively described by the parameters $\beta_S^U$, $\beta_S^T$ and $\beta_R$.

We assume that the expected time that an adult acquires new sex partners in Botswana is on average 34 years. This is exactly the difference in years between the upper (49 years) and the lower (15 years) adult age group that we consider. The inflow of at-risk susceptible adults, $\pi$, is chosen and calculated such that in the short term the total population is stationary in the absence of HIV. On the assumption that the population growth can be ignored over the short term, we obtained that $\pi$ is 49,300 per year. The contact rate was found to be 1.76 per year on the assumption that HIV prevalence is constant over the short term. In a similar way the contact parameter, $c$, is chosen and calculated such that in the absence of treatment ($g_s = 0$ and $\sigma_s = 0$) $Y_S^U$ is stationary in the short term. We calculated the per capita treatment rate per year ($\sigma_s$) from the projected number of patients that the Botswana ART program plans to treat over the next four years. The program plans to treat 85,000 patients by 2009 [1] (see Fig. 2). The treatment rate was found by assuming that it remains constant such that by the year 2009, Botswana will have 85,000 patients that have received ART. This procedure gave a constant per capita treatment rate of 0.050 per year. For Botswana, we assumed that $\mu^{-1}$ is 34 years (from 15 to 49 years old). We used the values of 10, 18 and 12 years respectively for $(v_S^U)^{-1}$, $(v_S^T)^{-1}$ and $(v_R)^{-1}$ representing the average progression time to AIDS [2, 3]. Thus, individuals that are on treatment remain sexually active for longer than individuals that are not on therapy, on average, for an additional 8 years. Since our predictions are valid for the short term to 2009, our results are robust to the values chosen for the average progression time to AIDS. We assumed that the yearly proportion of treated cases suspending ART, $g_S$, in Botswana is 0.1[4] and that, on average, drug resistance would develop in 3 to 5 years [5, 6, 7, 8, 9] (see the **Main Text** for further discussion).

The untreated and treated wild-type transmissibility (i.e., *fitness*) coefficients per partnership ($\beta_S^U$ and

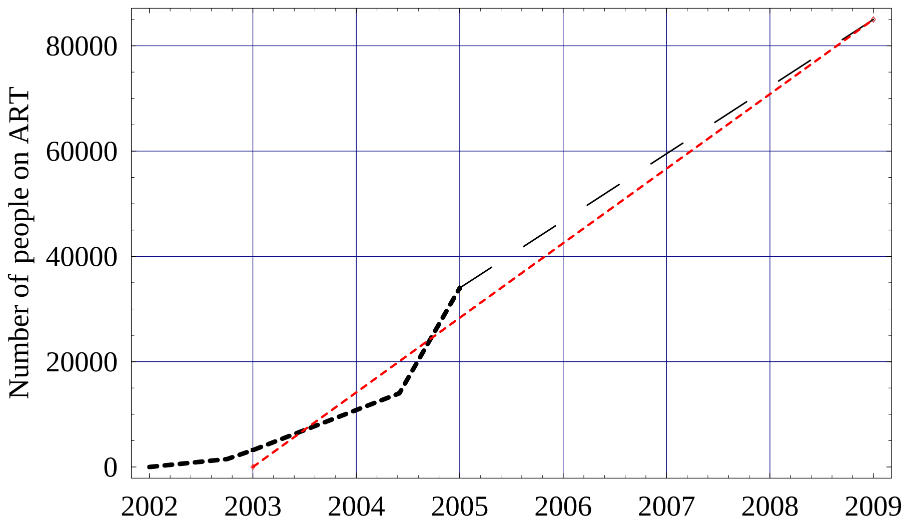


Figure 2: Empirical data from the Botswana's government treatment program are shown by the filled boxes. The aim of the Botswana ART program is to reach 85,000 patients by 2009 (black dashed line) [1]. The treatment used by our model uses the linear fit shown by the dashed gray line, this gives a constant per capita treatment rate of 0.050 per year.

$\beta_S^T$) were calculated to be 0.12 and 0.04 respectively [10, 11, 12]. Our values for $\beta_S^U$ and $\beta_S^T$ are consistent with those used by Wilson *et al.* [13] for South Africa for a similar drug regiment. We assume that $\beta_S^U$ lies in the range 0.1 to 0.15 [11]. We then calculate $\beta_S^T$ from $\beta_S^U$ using the following expression

$$\beta_S^T = (\alpha_T p_S + 1 - p_S)\beta_S^U, \tag{1.8}$$

where $\alpha_T$ is the reduction in HIV transmissibility (i.e., *fitness*) due to treatment-induced viral load reduction and $p_S$ is the fraction of treated individuals infected that achieve viral suppression on ART. Following Wilson *et al.*, the calculation of the drug-resistant transmissibility is obtained using the following expression

$$\beta_R = \alpha_R \beta_S^U, \tag{1.9}$$

where $\alpha_R$ is a proportionality constant. Relatively little is known about the fitness of resistant strains of HIV *in vivo*. In our paper we assumed $\alpha_R$ to be 0.25, 0.5 and 1.0 in different scenarios.

An alternative way of obtaining the values for transmissibility is to use the relationship that each logarithmic increase in viral load ($w$) is associated with an increase in the risk of transmission by a factor of 2.45 [12]. Explicitly, this relationship is formalized as

$$\beta(w) = \beta(\nu)2.45^{\log_{10} w/\nu}, \tag{1.10}$$

where $\beta(w)$ and $\beta(\nu)$ are transmissibilities as a function of viral loads $w$ and $\nu$.

### 1.3 The initial conditions

Our model is integrated starting from the initial conditions that no one is on treatment ($Y_S^T(0) = 0$). Therefore HIV drug resistance has not developed ($Y_R(0) = 0$). We assume that the HIV-infected wild-type population is at equilibrium with the susceptible population for our initial conditions. These equilibrium population values are given by:

$$X(0) = X^* = \frac{\pi}{c\beta_S^U - v_S^U} \tag{1.11}$$

$$Y_S^U(0) = (Y_S^U)^* = \frac{\pi(v_S^U - c\beta_S^U) + \pi\mu}{(v_S^U - c\beta_S^U)(v_S^U + \mu)}. \tag{1.12}$$

## 2 Stochastic formulation for the drug-resistant population dynamics

### 2.1 Stochastic vs. Deterministic

The ODEs formulated in section 1.1 can be numerically integrated to obtain the mean-field population evolution. As long as the populations present in each compartment are large, relative stochastic fluctuations are negligibly small. From the discussed initial conditions (Section 1.3), we are assuming that both the treated wild-type and the drug-resistant population begin their dynamics from zero. This means that relative stochastic fluctuations are important in their initial short-term dynamics. However, for the chosen parameters the initial inflow of individuals into the treated wild-type population is much larger than the inflow into the drug-resistant population. Thus, the time for which relative stochastic fluctuations are important is much greater for the drug-resistant population than for the treated wild-type population. Hence, the evolution of the wild-type population can be approximated by its mean-field dynamics at a much earlier time than the evolution of the drug-resistant population. For this reason, the forecast of the short-term dynamics of the drug-resistant population benefits from a stochastic analysis. This analysis provides not only the mean field evolution of the population numbers, but also the evolution of its variability. Our main interest is in the variability present in the prevalence and incidence patterns of the drug-resistant population. We therefore proceed by treating the drug-resistant population stochastically.

A description of the stochastic dynamics is often obtained via computational integration by means of Monte Carlo methods [18]. This would involve identifying the stochastic processes affecting the drug-resistant population dynamics and simulating the evolution discretely starting from the initial conditions. Then, in order to obtain the variability in the dynamics one would have to average over many independent ensemble realizations of this evolution. An alternative method is to try to solve the stochastic dynamics analytically by formulating and solving a Master equation [15, 16]. This is an attractive method for proceeding as it produces

exact equations to predict the variability in the dynamics. In problems in mathematical epidemiology, this can not always be done as transition rates present in the Master equation formulation are nonlinear functions of the population size. By assuming that the stochastic fluctuations have a predefined distribution (e.g., Gaussian or Log-Normal) this problem can be overcome to give an approximate solution via the method of moment closure [19]. However, in some problems the nonlinear transition rates can be approximated to linear transition rates that remain valid for the time interval of interest. Our stochastic formulation makes use of this latter approximation and we proceed in obtaining expressions for the variability of the dynamics given by time dependent functions of the variance and skewness of the process.

## 2.2 The Master equation

Eq. (1.4) can be used to formulate a birth-death-immigration Master equation [15, 16] for the drug-resistant population:

$$\begin{aligned} \partial P_k(t)/\partial t &= rY_S^T(t)[P_{k-1}(t) - P_k(t)] + \beta_R c X(t)/N(t)\,[(k-1)P_{k-1}(t) - kP_k(t)] \\ &- (v_R + \mu)\,[kP_k(t) - (k+1)P_{k+1}(t)]\ , \end{aligned} \tag{2.13}$$

where $P_k(t)$ denotes the probability that $Y_R(t) = k$. The set of equations described by (2.13) can be used to derive other equations describing the evolution of the moments of the probability distribution of $Y_R(t)$. By the standard approach, we multiply Eq.(2.13) throughout by $e^{\theta k}$ and sum over the $k$ states to obtain the master equation for the moment generating function $M(\theta, t)$. In turn, this can be expressed as an equation for the cumulant generating function defined as $K(\theta, t) = \log[M(\theta, t)]$.

$$\partial K(\theta,t)/\partial t = rY_S^T(t)(e^\theta - 1) + \left[\beta_R c X(t)/N(t)(e^\theta - 1) - (v_R + \mu)(1 - e^{-\theta})\right] \partial_s K(\theta, t) \tag{2.14}$$

The solution to the master equation allows us to extract cumulants of $Y_R(t)$:

$$\langle\langle Y_R(t)^n \rangle\rangle = \partial^n K(\theta,t)/\partial\theta^n|_{\theta=0}\ ; \tag{2.15}$$

the mean ($\langle\langle Y_R(t)\rangle\rangle$), variance ($\left\langle\left\langle Y_R(t)^2\right\rangle\right\rangle$)and the third central moment ($\left\langle\left\langle Y_R(t)^3\right\rangle\right\rangle$) are obtained for $n = 1$, $n = 2$ and $n = 3$ respectively. The skewness can be obtained from the third central moment ($\left\langle\left\langle Y_R(t)^3\right\rangle\right\rangle / \left\langle\left\langle Y_R(t)^2\right\rangle\right\rangle^{3/2}$). The transition rate $\beta_R c X(t)/N(t)$ is not constant and depends on the evolution of $Y_R(t)$. Therefore, as in most epidemic models this makes this transition rate in the master equation a nonlinear function of the population size. To proceed we note that our interest is in the early stages of the dynamics when the susceptible population and the HIV-infected wild-type population have only marginally changed from their initial equilibrium values. Therefore, for the chosen parameters, the approximation that $X(t)/N(t)$ stays constant at its equilibrium value is acceptable during the early stage of the evolution. This approximation is expressed as $X(t)/N(t) \sim (X)^*/[(X)^* + (Y_S^U)^*] = (X/N)^*$ [1]. Making use of this

[1]By comparing the mean evolution of the drug-resistant population predicted by the solution of the Master equation to the mean-field evolution given by solving the ODE (1.4), we find that this approximation produces at most a 9% error (see Sec. 2.5) at the sixth and final year of the predictions.

approximation allows us to solve for $Y_S^T(t)$ exactly:

$$Y_S^T(t) \quad = \quad \frac{\sigma_S(e^{\alpha t}-1)}{\alpha}(Y_S^U)^* = \frac{I}{r}(e^{\alpha t}-1). \tag{2.16}$$

where $\alpha = -(g_s + r + v_s^T + \mu)$ and $I = r\sigma_S(Y_S^U)^*/\alpha$. Making use of this solution and using $X(t)/N(t) \sim (X/N)^* = (v_S^U + \mu)/(c\beta_S^U)$ we can solve Eq. (2.14) analytically. By using the method of characteristics [17] we obtain three equations:

$$\tau = t,\ \ \dot{\theta} = -\{\beta(e^{\theta}-1) - \gamma(1-e^{-\theta})\},\ \ \dot{K} = I(e^{\alpha t}-1)(e^{\theta}-1), \tag{2.17}$$

where $\beta = \beta_R c(X/N)^*$ and $\gamma = (v_R+\mu)$. Solving for $\theta$ and letting our initial constant characteristic $f = \theta(0)$ we obtain

$$e^f = \frac{\gamma(e^{\theta}-1) - (\beta e^{\theta} - \gamma)e^{(\gamma-\beta)t}}{\beta(e^{\theta}-1) - (\beta e^{\theta}-\gamma)e^{(\gamma-\beta)t}}, \tag{2.18}$$

which can be re-written in the form

$$e^{\theta} - 1 = \frac{(\gamma-\beta)(e^f-1)e^{(\gamma-\beta)t}}{\beta(e^f-1)e^{(\gamma-\beta)t} - e^f\beta + \gamma}. \tag{2.19}$$

Substituting this expression in the equation for $\dot{K}$ (in (2.17)) gives us

$$K(\theta,t) - K(\theta,0) = I\int_0^t (e^{\alpha s}-1)\frac{(\gamma-\beta)(e^f-1)e^{(\gamma-\beta)s}}{\beta(e^f-1)e^{(\gamma-\beta)s} - e^f\beta+\gamma}ds. \tag{2.20}$$

This integral can be evaluated by appropriate substitutions. Finally, we use our initial condition of $P_k(0) = \delta_{k,Y_R(0)}$ which gives $K(\theta,0) = fY_R(0)$ and we obtain our solution as

$$\begin{aligned} K(\theta,t) \quad = \quad & Y_R(0)\log\left[\frac{\gamma(e^{\theta}-1) - (\beta e^{\theta}-\gamma)e^{(\gamma-\beta)t}}{\beta(e^{\theta}-1) - (\beta e^{\theta}-\gamma)e^{(\gamma-\beta)t}}\right] + \frac{I}{\beta}\log\left[\frac{(\beta-\gamma)e^{(\gamma-\beta)t}}{\beta(e^{\theta}-1) - (\beta e^{\theta}-\gamma)e^{(\gamma-\beta)t}}\right] \\ & + I\frac{(\beta-\gamma)e^{(\beta-\gamma)t}}{(\alpha+\gamma-\beta)(e^{\theta}\beta-\gamma)}\left\{e^{(\alpha-\beta+\gamma)t}(e^{\theta}-1)F_1(\theta) - (e^{\theta}-1)F_2(\theta,t)\right\}. \end{aligned} \tag{2.21}$$

Here,

$$F_1(\theta) \quad = \quad F\left[\frac{\alpha}{(\gamma-\beta)}+1, 1, \frac{\alpha}{(\gamma-\beta)}+2, \frac{\beta(e^{\theta}-1)}{(e^{\theta}\beta-\gamma)}\right], \tag{2.22}$$

$$F_2(\theta,t) \quad = \quad F\left[\frac{\alpha}{(\gamma-\beta)}+1, 1, \frac{\alpha}{(\gamma-\beta)}+2, e^{(\beta-\gamma)t}\frac{\beta(e^{\theta}-1)}{(e^{\theta}\beta-\gamma)}\right], \tag{2.23}$$

where $F[a,b,c,d]$ is the hypergeometric function.

## 2.3 Solution for the Mean, Variance and Skewness

By using Eq. (2.15), Eq. (2.21) and our initial condition of no drug-resistant cases ($Y_R(0) = 0$) we obtain all the cumulants of $Y_R(t)$. The mean is given by

$$\partial_{\theta}[K(\theta,t)]|_{\theta=0} = Ie^{(\beta-\gamma)t}\left(\frac{e^{-(\beta-\gamma)t}-1}{(\gamma-\beta)} - \frac{e^{(\alpha-\beta+\gamma)t}-1}{(\alpha+\gamma-\beta)}\right), \tag{2.24}$$

our second central moment (variance) is given by

$$\partial_\theta^2[K(\theta,t)]\big|_{\theta=0} = \frac{Ie^{(\beta-\gamma)t}}{(\beta-\gamma)^2}\left\{\left[\gamma(e^{(\alpha-\beta+\gamma)t}-1)+\beta(e^{(\beta-\gamma)t}-1)\right]+\frac{\alpha}{(\alpha+\gamma-\beta)(\alpha-2\beta+2\gamma)}\times\right.$$
$$\left.\left[e^{(\alpha-\beta+\gamma)t}[\beta^2+\beta\gamma-\gamma(\alpha+2\gamma)]-[(2-e^{(\beta-\gamma)t})\beta+2\gamma](\beta-\gamma)-\alpha[(e^{(\beta-\gamma)t}-1)\beta-\gamma]\right]\right\}; \quad (2.25)$$

and our third central moment is given by

$$\partial_\theta^3[K(\theta,t)]\big|_{\theta=0} = \frac{Ie^{(\beta-\gamma)t}}{(\alpha-2\beta+2\gamma)(\alpha-3\beta+3\gamma)}\left\{e^{(\alpha-\beta+\gamma)t}[\alpha^2+6\gamma(\beta+\gamma)+\alpha(\beta+5\gamma)]-\right.$$
$$(\beta-\gamma)^{-2}\left[6(\beta-\gamma)^2[\beta^2(1-3e^{(\beta-\gamma)t}+2e^{2(\beta-\gamma)t})-\beta\gamma(3e^{(\beta-\gamma)t}-4)+\gamma^2]+\right.$$
$$\alpha^2[\beta^2(1-6e^{(\beta-\gamma)t}+6e^{2(\beta-\gamma)t})-2\beta\gamma(3e^{(\beta-\gamma)t}-2)+\gamma^2]- \quad (2.26)$$
$$\left.\left.\alpha(\beta-\gamma)[\beta^2(5-24e^{(\beta-\gamma)t}+18e^{2(\beta-\gamma)t})-4\beta\gamma(6e^{(\beta-\gamma)t}-5)+5\gamma^2]\right]\right\}.$$

## 2.4 Solution for the Cumulative Incidence

From the solution of the Master equation we can also extract the cumulant generating function for the cumulative incidence of transmitted drug resistance ($T(\theta,t)$). To obtain this we need to solve the following partial differential equation that relates the rate of change in transmitted incidence to the prevalence of drug resistance in the population:

$$\partial_t T(\theta,t) = \beta_R c(X/N)^*(e^\theta-1)\partial_\theta K(\theta,t). \quad (2.27)$$

Therefore, the generating function for the cumulative transmitted drug resistance incidence between times $t_1$ and $t_2$ is given by:

$$T(\theta,t_2)-T(\theta,t_1) = \beta_R c(X/N)^*(e^\theta-1)\int_{t_1}^{t_2}\partial_\theta K(\theta,t)dt. \quad (2.28)$$

By taking the first, second and third derivatives of (2.28) with respect to $\theta$ and evaluating these at $\theta=0$ we obtain expressions for the dynamics of the mean, variance and skewness of the cumulative transmitted drug-resistant population in the interval $t_2-t_1$. We use these expressions in our main article to produce the probabilistic forecasts in the growth of the newly infected transmitted drug-resistant population for different parameter sets. In our manuscript we used time intervals $t_{n+1}-t_n$ each equal to three months.

## 2.5 Solution of the Master Equation versus the numerical integration of the ODEs

We mentioned in Sec. 2.1 that in our stochastic formulation we make use of an approximation whereby the nonlinear transition rates describing the infectious process is replaced by a linear transition rate. This approximation will stay valid initially and only for a short period. Therefore, it is of interest to check how the mean-field predictions obtained by solving the Master equation compare to those obtained by a numerical

integration of the ODE dynamics of Eqs.(1.1) - (1.4) for the duration of the dynamics of interest. The percentage error ($Er[S]$) made by the mean-field solution is given by

$$Er[S] = \frac{S_{\text{stochastic}} - S_{\text{numerical}}}{S_{\text{numerical}}}, \tag{2.29}$$

where $S_{\text{stochastic}}$ represents the mean-field solution of the dynamic variable $S$ obtained from the master equation and $S_{\text{numerical}}$ represents the corresponding numerical solution. Fig. 3 shows a plot for $Er[Y_R(t)]$ (shown in blue) and $Er[Y_R(t)/N(t)]$ (shown in red). Therefore, we compute the percentage difference of both the mean-field solution for $Y_R(t)$ and $Y_R(t)/N(t)$ to that obtained by numerical integration of the ODEs. The numerical integration was done using *Mathematica 5.1* by the default method that automatically switches between backward differentiation formulas and Adams multistep methods, depending on stiffness of the ODEs. We see from Fig. 3 that the error in $Y_R(t)$ (shown in blue) increases linearly with time from zero to 6% by 2009. This means that our predictions for the adult drug-resistant population overestimate the

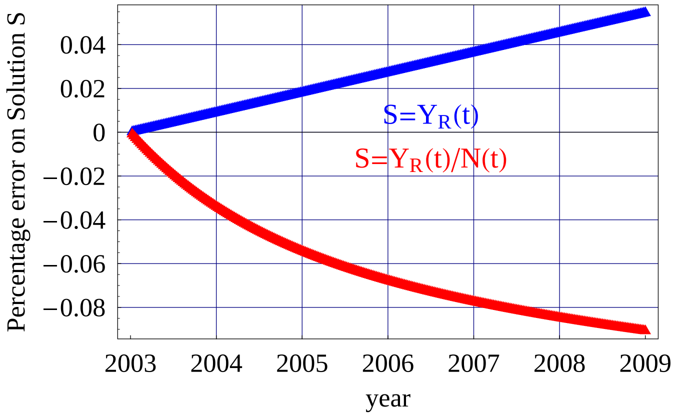


Figure 3: Percentage error of the mean-field solution for the prevalence of the drug-resistant adults obtained via the Master equation compared to the numerical integration of the deterministic equations of the model (Eqs.(1.1) - (1.4)). The red curve shows the error made by the drug-resistant prevalence expressed as a percentage of the total population (the error of $Y_R(t)/N(t)$). The blue curve shows the error in terms of absolute number drug-resistant adults (the error in $Y_R(t)$).

numerical solution by at most 6% in terms of absoulte numbers. However, since the numerical solution for $N(t)$ is a increasing function with time during the period (2003 to 2009) the error made by our predictions for the prevalence of drug-resistant adults underestimates the numerical solution by 9% by 2009. These error may seem large. However, considering parameter uncertainty that enter Eqs.(1.1) - (1.4), this is acceptable for obtaining an idea of the magnitude of stochastic fluctuations in the short-term dynamics.

# References


[1] Khan AB (2003) *The National HIV/AIDS Strategic Framework 2003-2009.* http://hivinsite.ucsf.edu/pdf/cr09-bc-01.pdf.

[2] Barbour JD, Hecht FM, Wrin T, Segal MR, Ramstead CA, Liegler TJ, Busch MP, Petropoulos CJ, Hellmann NS, Kahn JO & Grant, RM (2004) Higher CD4+ T cell counts associated with low viral pol replication capacity among treatment-naive adults in early HIV-1 infection. J Infect Dis 190: 251-6.

[3] Porter K & Zaba B (2004) The empirical evidence for the impact of HIV on adult mortality in the developing world: data from serological studies. AIDS 18 Suppl 2: S9-S17.

[4] Coetzee D, Hildebrand K, Boulle A, Maartens G, Louis F, Labatala V, Reuter H, Ntwana N & Goemaere E (2004) Outcomes after two years of providing antiretroviral treatment in Khayelitsha, South Africa. AIDS 18: 887-95.

[5] Jourdain G, Ngo-Giang-Huong N, Le Coeur S, Bowonwatanuwong C, Kantipong P, Leechanachai P, Ariyadej S, Leenasirimakul P, Hammer S & Lallemant M (2004) Intrapartum exposure to nevirapine and subsequent maternal responses to nevirapine-based antiretroviral therapy. N Engl J Med 351: 229-40.

[6] Richman DD, Morton SC, Wrin T, Hellmann N, Berry S, Shapiro MF & Bozzette SA (2004) The prevalence of antiretroviral drug resistance in the United States. AIDS 18: 1393-401.

[7] Tang JW & Pillay D. (2004) Transmission of HIV-1 drug resistance. J Clin Virol 30: 1-10.

[8] Vergne L, Kane CT, Laurent C, Diakhate N, Gueye NF, Gueye PM, Sow PS, Faye MA, Liegeois F, Ndir A, Laniece I, Peeters M, Ndoye I, Mboup S & Delaporte E (2003) Low rate of genotypic HIV-1 drug-resistant strains in the Senegalese government initiative of access to antiretroviral therapy. AIDS 17 Suppl 3: S31-8.

[9] Weidle PJ, Malamba S, Mwebaze R, Sozi C, Rukundo G, Downing R, Hanson D, Ochola D, Mugyenyi P, Mermin J, Samb B & Lackritz E (2002) Assessment of a pilot antiretroviral drug therapy programme in Uganda: patients' response, survival, and drug resistance. Lancet 360: 34-40.

[10] French M, Amin J, Roth N, Carr A, Law M, Emery S, Drummond F & Cooper D (2002) Randomized, open-label, comparative trial to evaluate the efficacy and safety of three antiretroviral drug combinations including two nucleoside analogues and nevirapine for previously untreated HIV-1 Infection: the OzCombo 2 study. HIV blin Trials 3: 177-85

[11] Gray RH, Wawer MJ, Brookmeyer R, Sewankambo NK, Serwadda D, Wabwire-Mangen F, Lutalo T, Li X, vanCott T & Quinn TC (2001) Probability of HIV-1 transmission per coital act in monogamous, heterosexual, HIV-1-discordant couples in Rakai, Uganda. Lancet 357: 1149-53.

[12] Quinn TC, Wawer MJ, Sewankambo N, Serwadda D, Li C, Wabwire-Mangen F, Meehan MO, Lutalo T & Gray RH (2000) Viral load and heterosexual transmission of human immunodeficiency virus type 1. Rakai Project Study Group. N Engl J Med 342: 921-9.

[13] Wilson D.P., Kahn J.O. & Blower S.M. (2006) Predicting the epidemiological impact of antiretroviral allocation strategies in KwaZulu-Natal: The effect of the urban-rural divide. PNAS 103(38):14228-14233.

[14] Blower S.M., Gershengorn H.B. & Grant R.M. (2000) A tale of two futures: HIV and antiretroviral therapy in San Francisco. Science 287: 650-4.

[15] Yule G.U., and Kendall M.G. (1948) An introduction to the theory of statistics, London: Charles Griffin.

[16] Bailey N.T.J. (1990) The Elements of Stochastic Processes with Applications to the Natural Sciences, John Wiley & Sons, New York.

[17] Vvedensky D. D., (1992) Partial Differential Equations (with Mathematica), Addison-Wesley.

[18] Gillespie D.T.A. (1976) general method for numerically simulating the stochastic time evolution of coupled chemical reactions, J. Comput. Phys. 22, 403-34.

[19] Lloyd A. L. (2004) Estimating variability in models for recurrent epidemics: assessing the use of moment closure techniques, Theor. Popul. Bio. 65, 49-65.
